# Aβ42 Mutants with Different Aggregation Profiles Induce Distinct Pathologies in *Drosophila*

**DOI:** 10.1371/journal.pone.0001703

**Published:** 2008-02-27

**Authors:** Koichi Iijima, Hsueh-Cheng Chiang, Stephen A. Hearn, Inessa Hakker, Anthony Gatt, Christopher Shenton, Linda Granger, Amy Leung, Kanae Iijima-Ando, Yi Zhong

**Affiliations:** 1 Cold Spring Harbor Laboratory, Cold Spring Harbor, New York, United States of America; 2 Laboratory of Neurodegenerative Diseases and Gene Discovery, Farber Institute for Neurosciences, Thomas Jefferson University, Philadelphia, Pennsylvania, United States of America; 3 Laboratory of Neurogenetics and Protein Misfolding Diseases, Farber Institute for Neurosciences, Thomas Jefferson University, Philadelphia, Pennsylvania, United States of America; 4 Department of Biochemistry and Molecular Biology, Thomas Jefferson University, Philadelphia, Pennsylvania, United States of America; 5 Department of Neurobiology and Behavior, State University of New York, Stony Brook, New York, United States of America; Swiss Federal Institute of Technology Lausanne, Switzerland

## Abstract

Aggregation of the amyloid-β-42 (Aβ42) peptide in the brain parenchyma is a pathological hallmark of Alzheimer's disease (AD), and the prevention of Aβ aggregation has been proposed as a therapeutic intervention in AD. However, recent reports indicate that Aβ can form several different prefibrillar and fibrillar aggregates and that each aggregate may confer different pathogenic effects, suggesting that manipulation of Aβ42 aggregation may not only quantitatively but also qualitatively modify brain pathology. Here, we compare the pathogenicity of human Aβ42 mutants with differing tendencies to aggregate. We examined the aggregation-prone, EOFAD-related Arctic mutation (Aβ42Arc) and an artificial mutation (Aβ42art) that is known to suppress aggregation and toxicity of Aβ42 *in vitro*. In the *Drosophila* brain, Aβ42Arc formed more oligomers and deposits than did wild type Aβ42, while Aβ42art formed fewer oligomers and deposits. The severity of locomotor dysfunction and premature death positively correlated with the aggregation tendencies of Aβ peptides. Surprisingly, however, Aβ42art caused earlier onset of memory defects than Aβ42. More remarkably, each Aβ induced qualitatively different pathologies. Aβ42Arc caused greater neuron loss than did Aβ42, while Aβ42art flies showed the strongest neurite degeneration. This pattern of degeneration coincides with the distribution of Thioflavin S-stained Aβ aggregates: Aβ42Arc formed large deposits in the cell body, Aβ42art accumulated preferentially in the neurites, while Aβ42 accumulated in both locations. Our results demonstrate that manipulation of the aggregation propensity of Aβ42 does not simply change the level of toxicity, but can also result in qualitative shifts in the pathology induced *in vivo*.

## Introduction

The amyloid-β-42 (Aβ42) peptide has been suggested to play a central role in the pathogenesis of Alzheimer's disease (AD), a devastating, and currently incurable, neurodegenerative disorder [1]. Aggregation of Aβ42 peptide in the brain parenchyma is a pathological hallmark of AD [2]. Genetic studies of early-onset familial AD (EOFAD) provide a strong causative link between Aβ42 and AD [3], and some mutations in the Aβ peptide promote amyloid fibril formation [4], [5]. These data suggest that Aβ42 aggregation might be involved in AD pathogenesis [6], and Aβ42 aggregation is therefore an attractive target for therapeutic intervention in AD [7].

*In vitro*, the neurotoxicity of Aβ42 has been often correlated with the tendency of Aβ42 to aggregate [8], [9]. However, recent evidence indicates that Aβ42 can form a variety of misfolded structures, including multiple monomer conformers, different types of prefibrillar assemblies, and structurally distinct amyloid fibrils, and that such structural polymorphisms may mediate the diverse toxic effects of Aβ42 [10]–[13]. These results suggest that manipulation of Aβ42 aggregation *in vivo* may not simply change the magnitude of toxicity, but also qualitatively modify its pathogenic effects.

We have previously shown that expression of the human Aβ42 peptide in *Drosophila* brains induces age-dependent memory defects, locomotor dysfunction, and neurodegeneration accompanied by Aβ42 deposits [14]. Using this model system, we investigated the correlation between the aggregation tendencies of Aβ42 and memory defects, as well as neurodegeneration, through genetic manipulation of Aβ42 aggregation. We demonstrated that manipulation of the aggregation propensity of Aβ42 qualitatively as well as quantitatively modified the pathogenicity of Aβ42 *in vivo*.

## Results and Discussion

**Figure 1:**
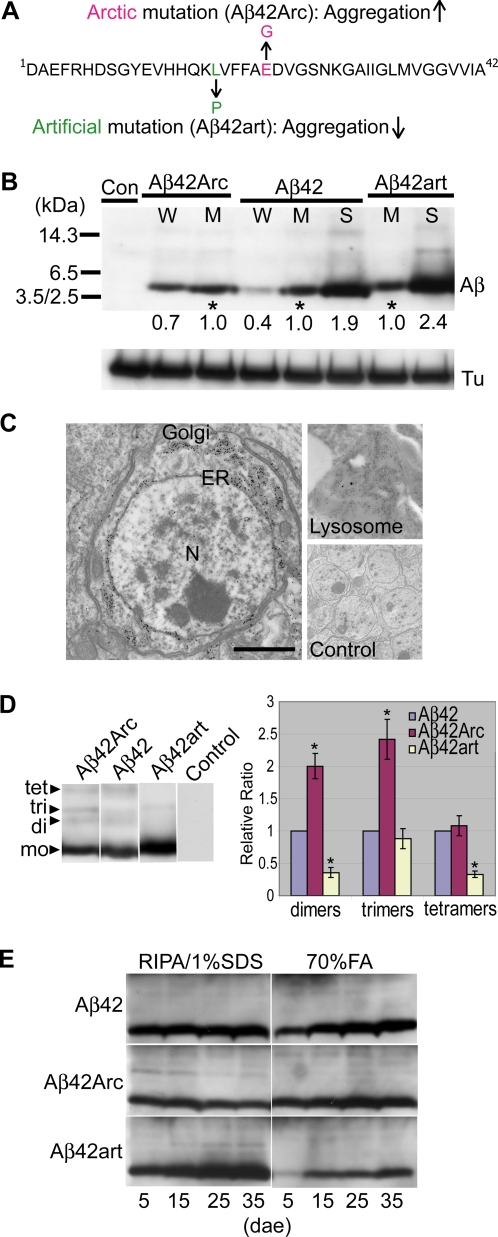
Expression, distribution, aggregation, and accumulation profiles of mutant Aβ42 peptides in fly brains. A, Sequences of Aβ42, Aβ42Arc, and Aβ42art. B, Expression levels of Aβ in independent transgenic lines (W; weak, M; moderate, S: strong expression) at 1-2dae (top panel, Aβ42), when accumulation of each Aβ in the insoluble fraction was minimum, were compared, and relative ratios were shown below each lane and in Table S1 (n = 3). Asterisks indicate the fly lines primarily used in this study. *elav-Gal4^c155^* flies were used as control. Tubulin was used as a loading control (bottom panel: Tu). C, ImmunoEM detection of Aβ42 in the endoplasmic reticulum (ER) and Golgi, as well as a lysosome. Gold particles are absent in the control (Control). N: nucleus, Scale bar: 1 µm. Neurons in Kenyon cell region of Aβ42 fly brains at 25dae were analyzed. D, Detection of dimers (di), trimers (tri) and tetramers (tet) in fly brains. The level of each oligomer was shown as a ratio relative to that of Aβ42. Asterisks indicate significant differences from Aβ42 (n = 3, P<0.05, Student's t-test). E, Age-dependent accumulation of Aβ peptides in detergent-soluble and insoluble fractions. The ages of the flies are indicated at the bottom.

The human Aβ42 with the Arctic mutation (E22G substitution, Aβ42Arc) (Figure 1A), which causes early onset familial AD (EOFAD) [4], is more aggregation-prone and toxic *in vitro* [5], [15] and accelerates the formation of amyloid deposits in the brains of AD model mice [16], [17]. In contrast, an artificial mutation, (L17P substitution, Aβ42art) (Figure 1A), suppresses amyloid fibril formation and toxicity *in vitro* [9], [18] and prevents the formation of amyloid deposits in *C. elegans* muscle [19].

A signal sequence was fused to the N-terminus of each Aβ [14], to target the peptide to the secretory pathway. Multiple transgenic lines carrying a *UAS-Aβ42*, *UAS-Aβ42Arc*, or *UAS-Aβ42art* transgene were established. Expression of each Aβ in the brain was driven by the pan-neuronal *elav-Gal4^c155^* driver [20]. Since *elav-Gal4* is on the X chromosome, male progeny expressed more Aβ peptide and developed stronger phenotypes than female progeny due to dosage compensation (data not shown). The results presented in this study are from male flies, unless otherwise indicated.

Western blot analysis detected monomeric forms of Aβ42, Aβ42Arc, and Aβ42art as 4 kDa signals (Figure 1B). Monomeric and oligomeric forms of Aβ42art migrated slower than those of Aβ42 due to an amino acid substitution (Figure 1B and D). Immunoprecipitation followed by mass spectrometry analysis confirmed that the fused signal peptide was correctly cleaved, and intact Aβ42, Aβ42Arc, and Aβ42art peptides were produced (Figure S1A–C). Immuno-electron microscopy (Immuno-EM) detected Aβ42 signals in the secretory pathway, including ER, Golgi, and lysosomes (Figure 1C), with minimal signals in the mitochondria and cytoplasm of neurons in the Kenyon cell region of Aβ42 fly brains. Aβ42Arc and Aβ42art peptides were also detected in the secretory pathway (data not shown). Secretion of Aβ peptides occurred in *Drosophila* cultured cells (Figure S2), and, in *Drosophila* brains, immuno-EM analysis occasionally detected Aβ42 accumulation in glial cells suggesting that Aβ42 peptides were secreted from neurons and then taken up by glial cells (Figure S3).

All Aβ peptides caused late-onset locomotor defects and premature death when expressed in neurons. Since the severity of these phenotypes positively correlated with the expression level of the peptides (Figure S4 and Table S1), we selected transgenic lines with similar expression levels (Figure 1B, asterisks) to characterize the accumulation profiles and the pathogenic effects of each Aβ peptide in the *Drosophila* brain.

To compare the ability of each Aβ peptide to form small oligomers, we quantified the levels of dimers (8 kDa), trimers (12 kDa), and tetramers (16 kDa), as detected by Western blotting (Figure 1D). This analysis revealed that Aβ42Arc formed 2-fold more dimers and trimers than did Aβ42, while Aβ42art formed 50% fewer dimers and tetramers. We also tested whether higher molecular weight Aβ oligomers were formed in fly brains with dot blot analysis using A11 antibody [21]. We did not observe A11-positive oligomers in any of Aβ42Arc, Aβ42 and Aβ42art brain lysates (Figure S5). During aging, Aβ42 and Aβ42Arc accumulated in the insoluble fraction of brain lysates (Figure 1E; extracted by 70% formic acid), with no significant accumulation in the soluble fraction (Figure 1E; extracted by RIPA/1%SDS). Accumulation of Aβ42Arc in the insoluble fraction was more aggressive than that of Aβ42 (Figure 1E, compare Aβ42 and Aβ42Arc in 5 days-after-eclosion (dae) flies). In contrast, Aβ42art strongly accumulated in the soluble fraction with greatly reduced accumulation in the insoluble fraction (Figure 1E). It should be noted that although age-dependent accumulation of Aβ42Arc in FA fraction from 5 to 25dae was clearly observed, the level of Aβ42Arc at 35dae was less than that at 25dae, presumably due to a progressive cell loss in Aβ42Arc fly brains (See below). These results demonstrate that Aβ42Arc is consistently more, and Aβ42art is significantly less, prone to aggregate *in vivo*.

**Figure 2:**
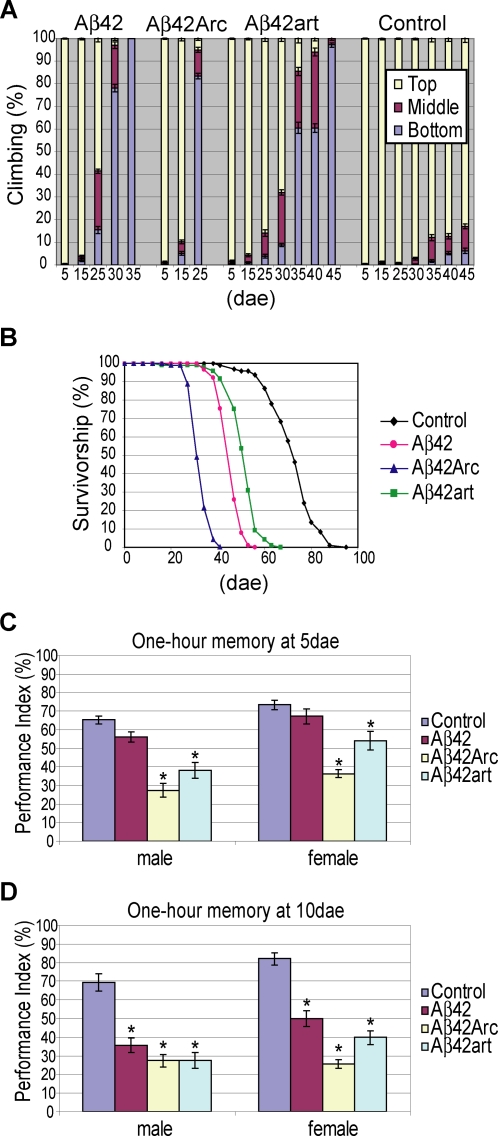
Behavioral defects in Aβ42, Aβ42Arc, and Aβ42art flies. A, Locomotor dysfunction. The percent of flies at the top (yellow), middle (magenta) or bottom (blue) of the vial at 10 seconds after knocking flies to the bottom are shown (average±SEM (n = 10)). B, Premature death. The percent survivorship was plotted against the age (dae). C and D, Memory defects. One hour memory was assessed by Pavlovian olfactory conditioning at 5 dae (C) and 10 dae (D). Asterisks indicate a significant difference from control (n = 6 or 8, α<0.05, Tukey-Kramer significant difference). Average memory scores±SEM are shown.

The severity of locomotor dysfunction and premature death phenotypes of the transgenic flies correlated well with the aggregation proneness of the Aβ peptides. Climbing ability was used to quantify locomotor activity [22]. Climbing disability in 80% of the flies occurred by 25, 35, and 45 dae in Aβ42Arc, Aβ42, and Aβ42art flies, respectively (Figure 2A). A similar tendency was observed for the premature death phenotype. The average lifespan of Aβ42Arc flies (32.9 dae) was shorter than that of Aβ42 flies (46.2 dae), while Aβ42art flies (51.4 dae) lived longer than Aβ42 flies (Figure 2B).

However, the onset of memory defects measured by Pavlovian olfactory classical conditioning [23] did not follow this simple trend. This assay was conducted with younger flies (5 or 10 dae), before the flies developed locomotor defects (Figure 2A). Data obtained from male and female flies are presented separately, since expression of Aβ is higher in males than in females, as a result of dosage compensation. For 5 dae flies in both male and female groups, Aβ42Arc flies showed the most severe 1 hour memory defects (memory scores were measured 1 hour after the training session), and Aβ42art flies were also defective (Figure 2C). In contrast, memory in Aβ42 flies was indistinguishable from control flies (Figure 2C). For 10 dae flies, all Aβ flies reached a similar level of memory defects in the male group (left panel in Figure 2D). In the female group, memory scores remained the lowest in Aβ42Arc flies, and both Aβ42art and Aβ42 flies showed similar defects (Figure 2D). A similar tendency was observed with the learning assay (learning scores were obtained immediately after training) (Figure S6). Of note, learning scores were normal in both 10 dae Aβ42 and Aβ42art female flies, indicating that these flies were specifically defective in short-term memory, the major clinical manifestation observed in patients in the early stages of AD [24]. The sensory motor activity of the flies, including sensing odors and electric shock, was indistinguishable from controls at 10 dae (Table S2), indicating that the observed defects can be interpreted as learning and memory defects.

**Figure 3:**
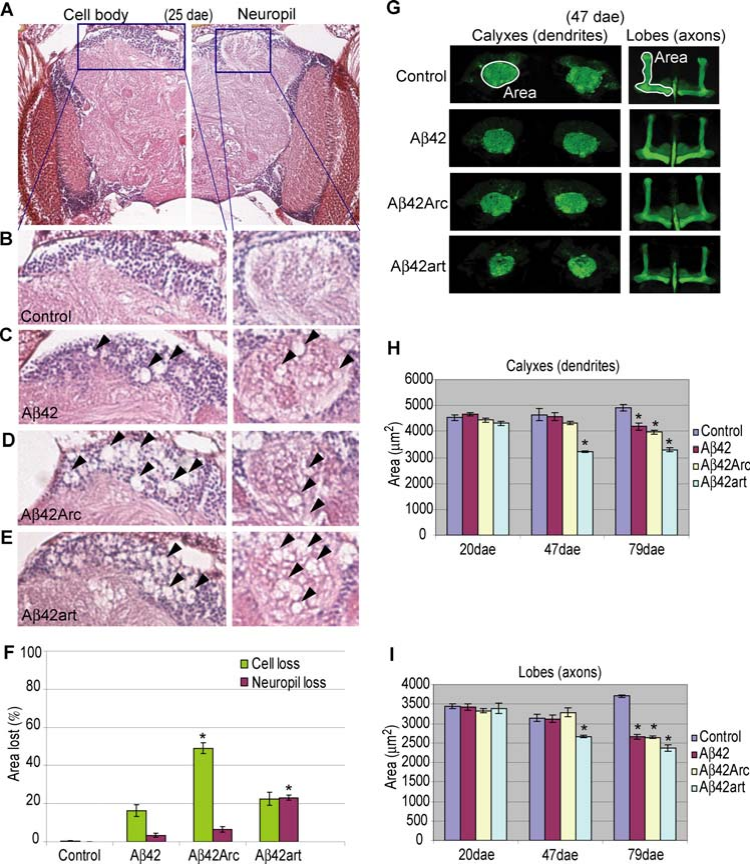
Cell body and neuropil degeneration in Aβ42, Aβ42Arc, and Aβ42art flies. A–E, Neurodegeneration in Aβ flies at 25 dae. The cell body and neuropil region in the mushroom body are enlarged. Arrowheads indicate neurodegeneration (C to E). F, Percentage of the area lost in the cell body (green) and neuropil (magenta) regions are shown as averages±SEM (n = 7–9 hemispheres). Asterisks indicate significant differences from Aβ42 (P<0.05, Student's t-test). G, Atrophy of Calyxes (dendrites) and Lobes (axons) in Aβ flies. H and I, Areas of Calyxes and Lobes were measured as indicated in (G) and presented as averages±SEM (n = 6 hemispheres). Asterisks indicate significant differences from control (P<0.05, Student's t-test). The ages of the flies are indicated at the bottom.

Remarkably, Aβ42, Aβ42Arc, and Aβ42art each induced distinct pathologies. Neurodegeneration in the Aβ fly brains was observed as a vacuolar appearance both in the cell body and neuropil regions. To quantify the area lost in these regions, we focused on the mushroom body structure, in which the cell bodies (Kenyon cell body), dendrites (Calyxes), and axon bundles (Lobes) were easily identified [25]. Our analysis revealed that, at 25 dae, Aβ42Arc fly brains showed more extensive cell loss than that in Aβ42 or Aβ42art brains (Figures 3A–F). However, the level of neuropil degeneration was greatest in Aβ42art flies (Figures 3A–F). The enhanced neuropil degeneration observed in Aβ42art flies was further confirmed by confocal analysis. In this assay, each Aβ42 peptide was preferentially expressed in mushroom body neurons using the *OK107-gal4* driver, and the structure of dendrites (Calyxes) and axons (Lobes) was visualized by co-expressed CD8-GFP[26] (Figure 3G). Quantification of the size of these structures revealed that Aβ42art induced earlier onset and more severe atrophy in both the dendrites (Calyxes) and axons (Lobes) among all Aβ flies (Figure 3G–I). The severity of the atrophy in Aβ42 and Aβ42Arc was similar. Observed differences were not due to differences in brain size (Figure S7).

**Figure 4:**
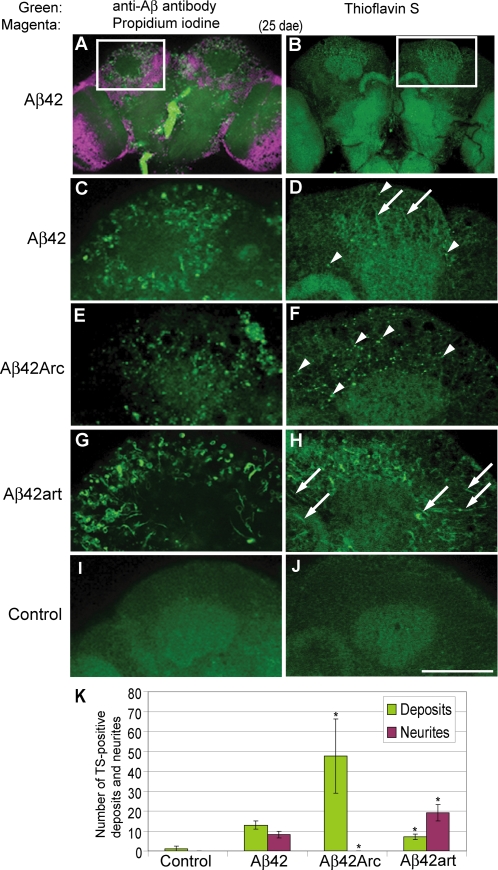
Distribution and aggregation of Aβ42, Aβ42Arc, and Aβ42art peptides in fly brains. A,C,E,G and I, Immunostaining of brains of 25 dae flies with anti-Aβ antibody (green). In (A), nuclei were stained with propidium iodide (magenta). B,D,F,H and J, Thioflavin S (TS) staining of brains of 25 dae flies. Arrowheads and arrows indicate TS-positive deposits and neurites, respectively. No signal was detected in the control (I, J). Scale bar in J: 50 µm. (C) and (D) are enlarged images of the boxed regions in (A) and (B), respectively. K, Numbers of TS-positive deposits and neurites were presented as averages±SD (n = 6 hemispheres). Asterisks indicate significant differences from Aβ42 (P<0.05, Student's t-test).

Immunostaining and Thioflavin S (TS) staining, which labels aggregated Aβ42, revealed that the degenerated structures in Aβ42, Aβ42Arc, and Aβ42art flies were closely correlated with the intraneuronal accumulation sites of each Aβ peptide. Aβ42Arc accumulated primarily in the cell soma as large deposits (Figure 4E, F, arrowheads in F), while Aβ42art was distributed primarily in the neurites (Figure 4G, H, arrows in H). Aβ42 was detected both in the cell body and in the neurites, but to a lesser extent than the mutants (Figure 4C, D, arrowheads and arrows in D). Quantification of TS-positive deposits and neurites in Aβ fly brains is shown in Figure 4K. These fly brains were negative for Congo-Red staining (Figure S8), and no intraneuronal amyloid fibrils were observed by electron microscopy, suggesting that the majority of TS-positive aggregates did not contain amyloid fibril structures. These distinct patterns of neurodegeneration and Aβ accumulation were confirmed in several independent transgenic lines (data not shown).

This study highlights that the complex toxicities of Aβ42 are associated with different aggregation propensities *in vivo*. First, the increase in Aβ42 aggregation proneness associated with the pathogenic Arctic mutation (E22G) correlated with more severe detrimental effects on memory, locomotor ability, and lifespan than those caused by Aβ42 (Figure 2). These data are consistent with the fact that Aβ42Arc causes EOFAD [4], and indicates that aggregation proneness contributes to Aβ42 toxicity *in vivo*. Second, an artificial mutation (L17P) that decreased Aβ42 aggregation proneness suppressed the toxicities toward locomotor function and lifespan, but caused earlier onset of memory defects (Figure 2), showing that not all pathogenic effects of Aβ42 correlate directly with aggregation proneness. Third, the differences in aggregation tendencies of Aβ42 and derivatives correlated with qualitative shifts in pathology in the fly brain, exemplified by distinct neurodegeneration patterns accompanying the different accumulation profiles of Aβ42 peptides (Figure 3 and 4). Importantly, these differences are not due to a difference in genetic background [27], since these Aβ flies were generated in the same genetic background.

Under physiological conditions, the Aβ42 peptides are generated from amyloid precursor protein (APP) by β- and γ-secretases in the secretory pathway including, the transgolgi-network, endosome-system, and plasma membrane [28]. Because *Drosophila* has no or very low endogeneous β-secretase activity [29], we used the artificial expression system to achieve high expression levels of Aβ42 in fly brains. In our transgenic flies, Aβ42 peptides were expressed in the ER and distributed to the late secretory pathway compartments, axons, dendrites, and presynaptic terminals, as well as secreted from neurons (Figure 1C, S2 and S3). Although the ER is not a major cellular site for Aβ generation under physiological conditions [28], several lines of evidence suggest that, under abnormal conditions, Aβ may be generated, retained, or recycled back to the ER and may induce ER stress [30]–[35]. Our fly models may recapitulate neuronal dysfunction and degeneration induced by such abnormal intracellular metabolisms of Aβ42. It would be also important to examine the effects of the Arctic (E22G) and artificial (L17P) mutations on intracellular distribution and toxicities of Aβ42 generated from the full-length APP.

In summary, our results lead us to predict two issues. First, the partial prevention of Aβ42 amyloidgenesis by aggregation inhibitors may result in qualitative shifts in the pathogenic effects of Aβ42. Second, the tendency of Aβ42, a natively unfolded polypeptide consist primarily of random-coil structure in their native and soluble states [36], [37], to aggregate may be affected by a combination of genetic [38], environmental [39], and aging factors [40], and the resultant Aβ42 conformers or species may contribute to the heterogeneous pathogenesis of AD [41]. The existence of different “Aβ species” has been recently verified both *in vitro* [12] and *in vivo* [42].

## Materials and Methods

### *Drosophila* genetics and stocks

cDNA fragments encoding the human Aβ42, Aβ42Arc, and Aβ42art peptides were amplified by PCR from human APP cDNA, fused to the rat pre-proenkephalin signal peptide, cloned into the pUAST *Drosophila* transformation vector and microinjected into fly embryos of the *w^1118^* (*isoCJ1*) genotype. Several transgenic lines for each Aβ construct were established. The flies were raised and maintained at 25°C, under conditions of 70% humidity and a 12 h:12 h light:dark cycle. The transgenic *UAS-CD8::GFP;;OK107* line was a kind gift from Dr. L. Luo [26]. *UAS-nlsGFP* and *elav-GAL4^c155^* flies were obtained from the Bloomington *Drosophila* Stock Center. For Pavlovian olfactory conditioning, the *elav-GAL4^c155^* line was outcrossed with *w^1118^* (*isoCJ1*) flies, an isogenic line, for 5 generations. Genotypes of flies used in this study are as follows: Control; *elav-GAL4^c155^/Y*, Aβ42; *elav-GAL4^c155^/Y;; UAS-Aβ42/+*, Aβ42Arc; *elav-GAL4^c155^/Y;; UAS-Aβ42Arc/+* and Aβ42art; *elav-GAL4^c155^/Y;; UAS-Aβ42art/+*. In figure 3G, H and I, flies' genotypes are as follows: Control; *w/Y;UAS-CD8::GFP/+;;OK107/+*, Aβ42; *w/Y; UAS-CD8::GFP/+;UAS-Aβ42/+;OK107/+*, Aβ42Arc; *w/Y; UAS-CD8::GFP/+;UAS-Aβ42Arc/+;OK107/+* and Aβ42art; *w/Y; UAS-CD8::GFP/+;UAS-Aβ42art/+;OK107/+*.

### Western blot and dot blot analysis

For sequential extractions, fly heads were homogenized in RIPA buffer (50 mM Tris-HCl, pH 8.0, 0.5% sodium deoxycholate, 1% Triton X-100, 150 mM NaCl) containing 1% SDS. Lysates were centrifuged at 100,000 g for 1h, and supernatants were collected (SDS-soluble fraction). SDS-insoluble pellets were further homogenized in 70% formic acid (Sigma) followed by centrifugation at 13,000 rpm for 20 min and the supernatants were collected (FA fraction). Formic acid was evaporated by Speed Vac (Savant, SC100) and protein was resuspended in dimethyl sulfoxiside (Sigma).

Protein extracts were immunoprecipitated with the anti-Aβ antibody 6E10 (Signet), separated on 10–20% Tris-Tricine gels (Invitrogen), and transferred to nitrocellulose membranes (Invitrogen). The membranes were boiled in phosphate buffered saline (PBS) for 3 min, blocked with 5% non-fat dry milk (Nestlé) and blotted with the 6E10 antibody or anti-tubulin antibody (Sigma). To quantify levels of expression of the Aβ peptide, heads from 1–2 dae flies were homogenized in Tris-Tricine sample buffer (Invitrogen), centrifuged at 13,000 rpm for 20 min and the supernatants were subjected to Western blotting, as described above. The signal intensity was quantified using ImageJ (NIH).

For dot blot analysis, fly heads were homogenized in 2% SDS followed by centrifugation at 13,000 rpm for 10 min. The supernatants were applied to a nitrocellulose membrane and air-dried. The membranes were blocked with 5% non-fat dry milk (Nestlé) and blotted with the A11 (Invitrogen) or 6E10 antibodies (Signet).

### Immunoprecipitation and mass spectrometric analysis

Two hundred Aβ fly heads were homogenized in 70% formic acid (Sigma) followed by centrifugation at 13,000 rpm for 20 min. The supernatant was evaporated using a Speed Vac (Savant, SC100) and protein was resuspended in dimethyl sulfoxiside (Sigma). The Aβ peptides were immunoprecipitated with the 6E10 antibody (Signet) and Protein-G Sepharose beads. The beads were washed three times with washing buffer (140 mM NaCl, 10 mM Tris-HCl, pH 8.0) containing 0.1% Octyl β-D-glucopyranoside, and the peptides were eluted with 70% formic acid and subjected to MALDI-TOF mass spectrometry.

### Climbing assay

Approximately 25 flies were placed in an empty plastic vial. The vial was gently tapped to knock the flies to the bottom and the number of flies at the top, middle, or bottom of the vial was scored after 10 seconds under red light (Kodak, GBX-2, Safelight Filter). Experiments were repeated more than three times, and a representative result was shown.

### Survival assay

Food vials containing 25 flies were placed on their sides at 25°C, under conditions of 70% humidity and a 12 h:12 h light:dark cycle. Food vials were changed every 2–3 days, and the number of dead flies was counted each time. At least four vials for each genotype were prepared. Experiments were repeated more than three times, and a representative result was shown.

### Pavlovian olfactory associative learning

Approximately 100 flies were trained by exposure to electroshock paired with one odour [octanol (OCT, 10^−3^(v/v)) or methylcyclohexanol (MCH, 10^−3^(v/v))] for 60 s and subsequent exposure to the other odour without electroshock for 60 s [23]. Immediately after training, learning was measured by allowing flies to choose between the two odours for 120 s. For one hour memory, trained flies were transferred to food vials, which were placed on their side in the dark at 25°C and 70% humidity, and tested after one hour. The performance index (PI) was calculated by subtracting the number of flies making the incorrect choice from those making the correct one, dividing by the total number of flies, and multiplying by 100. Absolute odour avoidance was quantified by a T-maze with one of the two odours (octanol [10^3^ (vol/vol)] or methylcyclohexanol [10^3^ (vol/vol)]) coming from one side and air from the other side. Naïve flies avoid odours, and the performance index was calculated by subtracting the number of flies that chose the odour side of the T-maze from those in the air side, dividing by the total number of flies and multiplying by 100. Electric shock reactivity was tested by putting approximately 100 flies in a T-maze having one arm with electric shock and one arm without electric shock. The performance index was calculated by subtracting the number of flies that chose the electric shock arm of the T-maze from those in the arm without shock, dividing by the total number of flies and multiplying by 100.

### Quantification of neurodegeneration

Heads were fixed in 4% paraformaldehyde (Electron Microscopy Sciences), processed to embed in paraffin blocks, and sectioned at a thickness of 6 µm. Sections were placed on slides, stained with haematoxylin and eosin (Vector), and examined by bright-field microscopy. To quantify neurodegeneration in the cell body and neuropil of the mushroom body structures, images of the sections which included the Kenyon cell body and/or Calyx were captured, and the area of the vacuoles in the Kenyon cell body or Calyx region was measured in each image. The ratio was calculated by dividing the sum of the vacuole areas by the total area of the Kenyon cell body or Calyx region. Seven to nine hemispheres from five flies were analyzed for each genotype. To quantify the atrophy of dendritic and axonal structures of the mushroom body neurons, the GFP signal in whole fly brains carrying *UAS-CD8::GFP;;OK107* was analyzed using confocal microscopy (Carl Zeiss LSM 510). The area of Calyxes (dendritic structures of the Kenyon cells), Lobes (axon bundles of the Kenyon cells) and the size of the brains was measured using LSM Image software. Six hemispheres from three flies were quantified for each genotype.

### Whole-mount immunostaining and Thioflavin S staining

Fly brains were dissected in cold PBS and fixed in PBS containing 4% paraformaldehyde (EMS), and then placed under vacuum in PBS containing 4% paraformaldehyde and 0.25% Triton X-100. After permeabilization with PBS containing 2% Triton X-100, the brains were treated with 70% formic acid (Sigma), and stained with a mouse monoclonal anti-Aβ antibody (Chemicon) followed by detection with biotin-XX goat anti-mouse IgG and streptavidin-Oregon Green 488 conjugate (Molecular Probes). Nuclei were counterstained with propidium iodide (Molecular Probes). To detect nuclei of glial cells, fly heads were stained with an anti-Repo antibody (DSHB) followed by detection with Texas Red goat anti-mouse IgG (Molecular Probes). The brains were analyzed using a confocal microscope (Carl Zeiss LSM 510). For Thioflavin S (TS) staining, the brains were permeabilized and incubated in 50% EtOH containing 0.1% TS (Sigma) overnight. After washing in 50% EtOH and PBS, the brains were analyzed using a confocal microscope. TS-positive deposits and neurites were quantified from six hemispheres from three flies per genotype.

### Congo-Red staining

Heads were fixed in 4% paraformaldehyde, processed to embed in paraffin blocks, and sectioned at a thickness of 10 µm. Sections were placed on slides and stained with an amyloid stain, Congo Red kit (Sigma), following the manufacuturer's protocol. Apple-green birefringence was examined by bright-field microscopy with a polarizing filter (Leica). Slides of human kidney tissue containing intracellular amyloid (Sigma) were processed at the same time as positive controls.

### Immuno-gold labelling and electron microscopy

Probosces were removed from decapitated heads, which were then immersion-fixed overnight in 4% glutaraldehyde and 2% paraformaldehyde in 0.1 M PBS. Samples were post-fixed 1 h in ferrocyanide-reduced osmium tetroxide (1% osmium tetroxide and 1.5% potassium ferrocyanide). Fixation was followed by dehydration in a graded alcohol series and infiltration with LR White resin (2 h in 50% LR White in ethanol and 24 h in 100% LR White) using constant rotation. After transferring the samples to gelatin capsules with fresh LR White resin, the samples were polymerized overnight at 60°C. Thin sections (100 nm) of Kenyon cells and neuropil regions of the mushroom body were collected on nickel grids (100 mesh, Veco-EMS). For immunogold labelling of Aβ42 transgenic and control fly heads, thin sections were first incubated for 2 minutes in 10% hydrogen peroxide for antigen retrieval, jet-rinsed in distilled water, and then placed on drops of 1% deacetylated BSA in PBS for 5 min. The grids were then transferred to drops of a rabbit antibody specific for human Aβ42 (Chemicon-Millipore) diluted 1∶10 in PBS and incubated for 2 h at room temperature. Unbound primary antibody was removed by rinsing the grids through 5 drops of PBS. Antibody was detected by incubating grids for 1 h in 10 nm colloidal gold conjugated goat anti-rabbit H&L (GE Healthcare) diluted 1∶10 in PBS. Grids were then rinsed in 10 drops of distilled water and air-dried. Thin sections were counterstained for 5 minutes in 3% uranyl acetate dissolved in 30% ethanol and then rinsed in distilled water.

### *Drosophila* S2 cell culture

*Drosophila* Schneider's cells (S2 cells) were maintained in Schneider's *Drosophila* Medium (Gibco) supplemented with 10% FBS (GEMINI) and an Antibiotic-Antimycotic mixture (Gibco). The cells were transiently transfected with *Actin-Gal4* and *UAS-Aβ* plasmid constructs using a calcium phosphate transfection kit (Invitrogen). Culture medium was replaced at 12 h post-transfection, and cells were cultured for an additional 24 h. The cells and culture medium were then harvested and subjected to immunoprecipitation followed by Western blot analysis as described above.

## Supporting Information

Figure S1 MS/IP analysis of Aβ peptides expressed in fly brains. Each Aβ peptide was immunoprecipitated using the anti-Aβ antibody and subjected to MALDI-TOF mass spectrometry. Aβ42 (A), Aβ42Arc (B), and Aβ42art (C) were each detected at their predicted mass.
(0.98 MB PDF)

Figure S2 Secretion of Aβ peptides expressed in Drosophila S2 cells. The levels of Aβ42 (blue), Aβ42Arc (magenta), and Aβ42art (green) in the culture medium were detected by Western blotting, normalized to intracellular Aβ levels, and shown as a ratio relative to that of Aβ42. Each Aβ peptide was secreted at different levels. Asterisks indicate a significant difference from Aβ42 (n = 3, P<0.05, Student's t-test).
(0.08 MB PDF)

Figure S3 Glial cells accumulate Aβ42 peptide produced in neurons in the Drosophila brain. ImmunoEM analysis detected Aβ42 (gold particles, arrowhead) in glial cells in brains of 25 dae flies with Aβ42 expression driven by elav-Gal4c155 (D). Scale bar in D: 1 µm. Confocal analysis revealed that elav-Gal4c155 does not drive the expression of transgene in glial cells. All nuclei of neurons in the fly brain were labeled by GFP fused to a nuclear localization signal driven by elav-Gal4c155 (A, green). The brain was counterstained with anti-Repo, a marker for Drosophila glial cells (B, magenta). The overlay image showed no significant overlap between the two signals (C). Scale bar in A, 50 µm.
(3.82 MB PDF)

Figure S4 Behavioral defects induced by the expression of Aβ42, Aβ42Arc, and Aβ42art peptides were dose dependent. A, C and E, Locomotor dysfunction in independent transgenic lines (W; weak, M; moderate, S; strong expression) of Aβ42 (A), Aβ42Arc (C), and Aβ42art (E). The percent of flies at the top (yellow), middle (magenta), or bottom (blue) of the vial at 10 seconds after knocking flies to the bottom are shown (average±SEM (n = 10)). B, D and F, The percent survivorship of independent transgenic lines (W, M, and S) of Aβ42 (B), Aβ42Arc (D), and Aβ42art (F) was plotted against the age (dae). The expression levels of Aβ peptides in all transgenic lines are shown in Figure 1B, and indicated as S (strong), M (moderate) or W (weak). The results are summarized in table S1. In the main text, the data from Aβ42 M, Aβ42Arc M and Aβ42art M (asterisks in Figure 1B) are presented.
(0.40 MB PDF)

Figure S5 No A11-positive Aβ oligomers in Aβ42, Aβ42Arc, and Aβ42art fly brains. Fly brain lysates (1 or 2 µl) were applied on membrane and probed with 6E10 (top) or oligomer-specific antibody, A11 (bottom). No specific signal was observed with A11 antibody (top, compare Control and Aβ42Arc, Aβ42 or Aβ42art), while 6E10 detected Aβ in fly brains (bottom, compare Control and Aβ42Arc, Aβ42 or Aβ42art). elav-Gal4c155 flies were used as control.
(1.61 MB PDF)

Figure S6 Learning defects in Aβ42, Aβ42Arc, and Aβ42art flies. Learning ability was assessed by Pavlovian olfactory conditioning at 10 dae. Asterisks indicate a significant difference from control (n = 6–8, α<0.05, Tukey-Kramer significant difference). Average learning scores±SEM are shown.
(0.04 MB PDF)

Figure S7 Brain sizes of control, Aβ42, Aβ42Arc, and Aβ42art flies were not significantly different. The thickness of fly brains was measured as indicated, and presented as average±SEM (n = 5 individual flies). The age of the flies is indicated at the bottom.
(0.62 MB PDF)

Figure S8 The majority of Aβ aggregates in fly brains are not stained by Congo-Red. Paraffin sections of brains of 25 dae control (B), Aβ42 (C), Aβ42Arc (D) and Aβ42art (E) flies were stained with Congo-Red. Sections from human kidney tissue containing intracellular amyloid were used as a positive control (A). Amyloid was observed as pink signals under bright field, and β-pleated structures were detected as birefringent apple-green signals using a polarizing filter (arrows in (A)). None of Aβ fly brains were stained with Congo-Red (C–E). The numerous vacuoles in (C–E) indicate neurodegeneration. For this analysis, transgenic lines with the highest expression of each Aβ peptide (Aβ42 S, Aβ42Arc M and Aβ42art S) were used.
(7.67 MB PDF)

Table S1 Summary of the characterization of Aβ transgenic lines. Asterisks (*) indicate transgenic lines primarily used in this study.
(0.08 MB PDF)

Table S2 Shock reactivity and olfactory acuity of transgenic flies at 10 dae. Aβ42, Aβ42Arc, and Aβ42art flies did not show any significant differences from control flies (n = 6, α<0.05, Tukey-Kramer significant difference). Average scores±SEM are shown. In MCH olfactory acuity of males, there were no differences relative to controls, but Aβ42Arc and Aβ42art flies were significantly different from each other (*).
(0.18 MB PDF)
