# Supplementary figures and images for: Aβ42 Mutants with Different Aggregation Profiles Induce Distinct Pathologies in Drosophila
Source: PLoS One. 2008 Feb 27;3(2):e1703. doi: 10.1371/journal.pone.0001703 (PMC2250771; doi:10.1371/journal.pone.0001703)

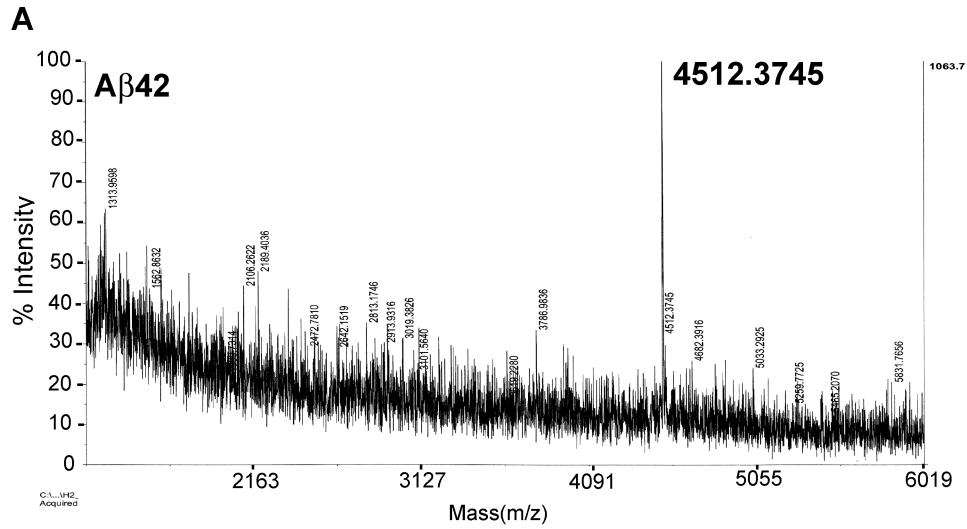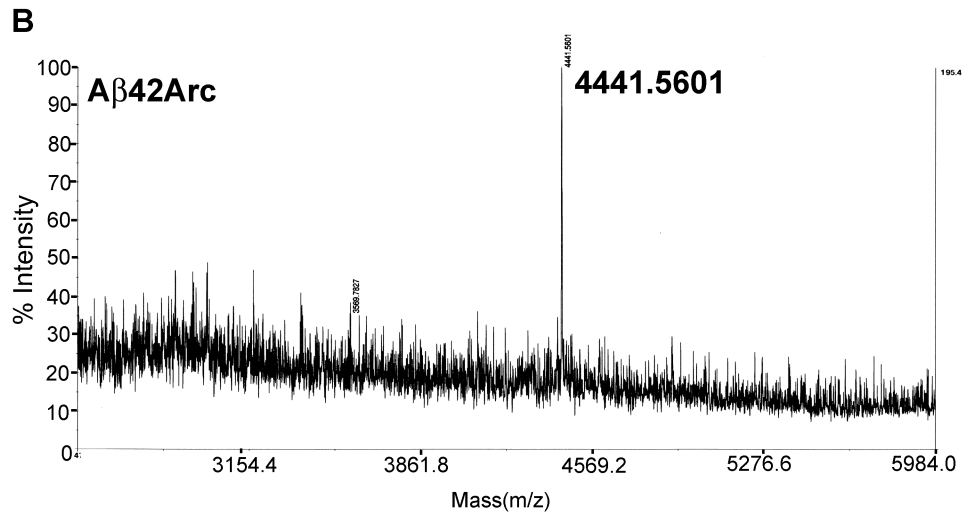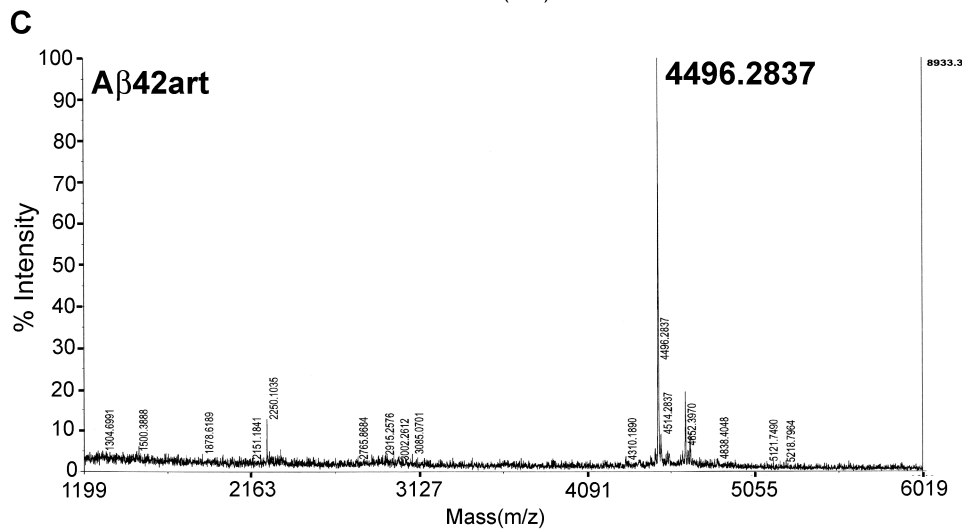

Supplement: Figure S1 — MS/IP analysis of Aβ peptides expressed in fly brains. Each Aβ peptide was immunoprecipitated using the anti-Aβ antibody and subjected to MALDI-TOF mass spectrometry. Aβ42 (A), Aβ42Arc (B), and Aβ42art (C) were each detected at their predicted mass. (0.98 MB PDF) [file pone.0001703.s001.pdf]

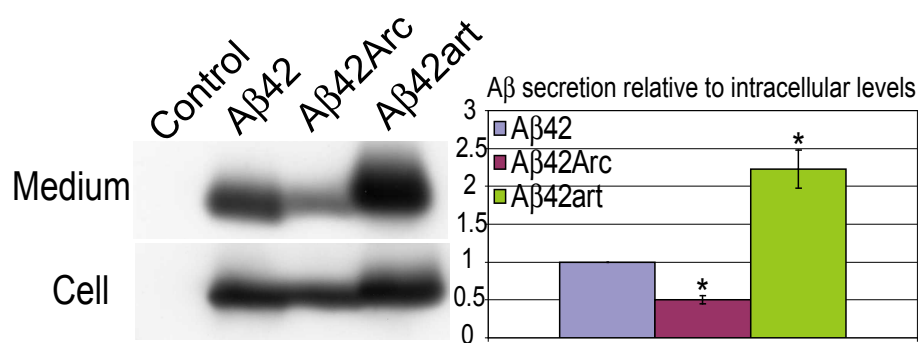

Supplement: Figure S2 — Secretion of Aβ peptides expressed in Drosophila S2 cells. The levels of Aβ42 (blue), Aβ42Arc (magenta), and Aβ42art (green) in the culture medium were detected by Western blotting, normalized to intracellular Aβ levels, and shown as a ratio relative to that of Aβ42. Each Aβ peptide was secreted at different levels. Asterisks indicate a significant difference from Aβ42 (n = 3, P<0.05, Student's t-test). (0.08 MB PDF) [file pone.0001703.s002.pdf]

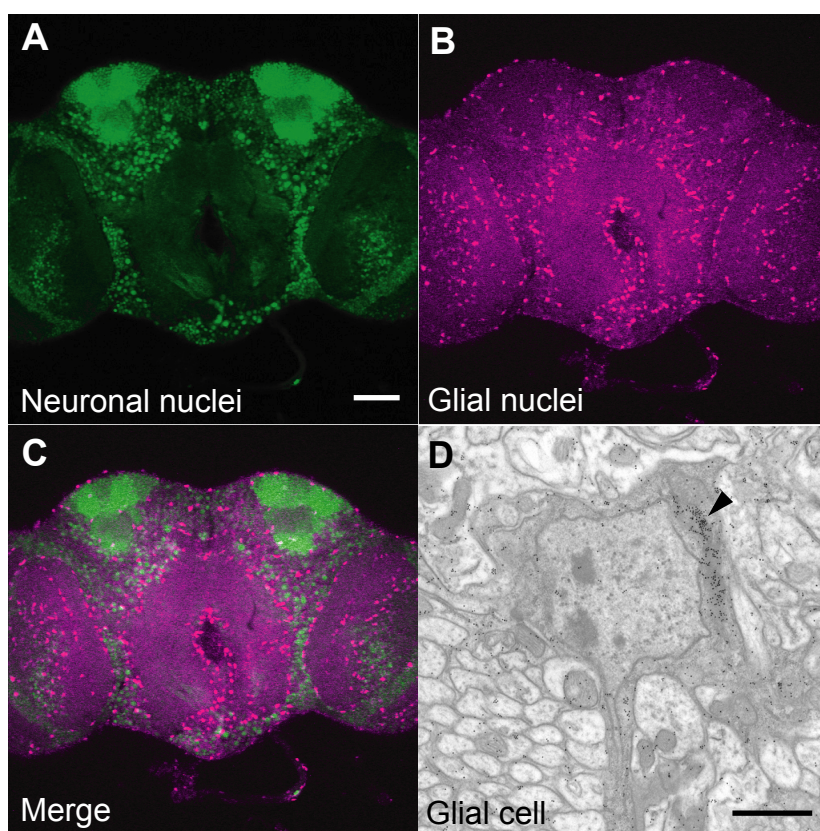

Supplement: Figure S3 — Glial cells accumulate Aβ42 peptide produced in neurons in the Drosophila brain. ImmunoEM analysis detected Aβ42 (gold particles, arrowhead) in glial cells in brains of 25 dae flies with Aβ42 expression driven by elav-Gal4c155 (D). Scale bar in D: 1 µm. Confocal analysis revealed that elav-Gal4c155 does not drive the expression of transgene in glial cells. All nuclei of neurons in the fly brain were labeled by GFP fused to a nuclear localization signal driven by elav-Gal4c155 (A, green). The brain was counterstained with anti-Repo, a marker for Drosophila glial cells (B, magenta). The overlay image showed no significant overlap between the two signals (C). Scale bar in A, 50 µm. (3.82 MB PDF) [file pone.0001703.s003.pdf]

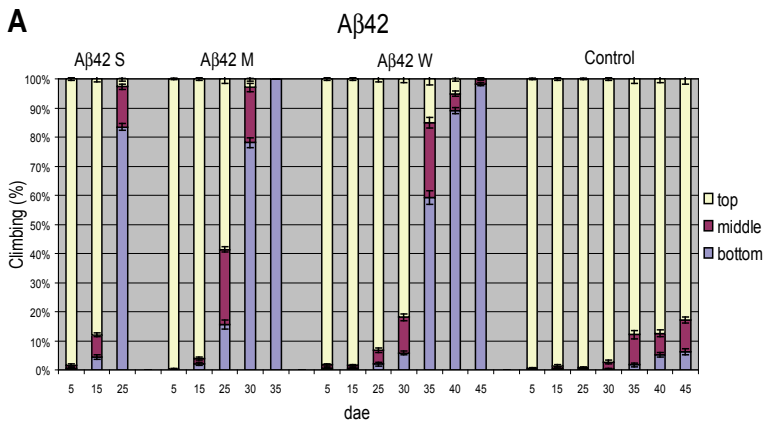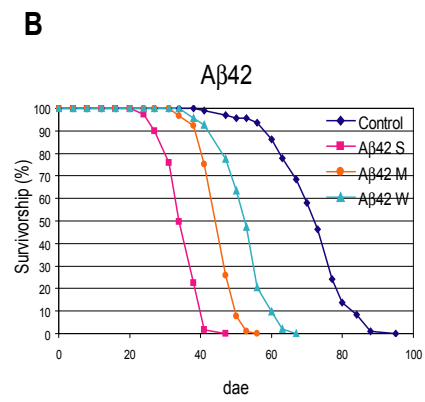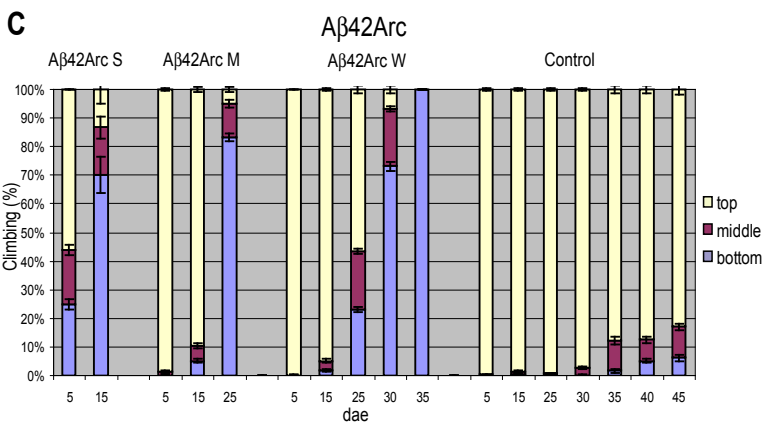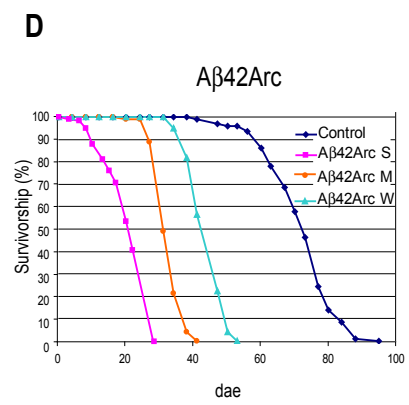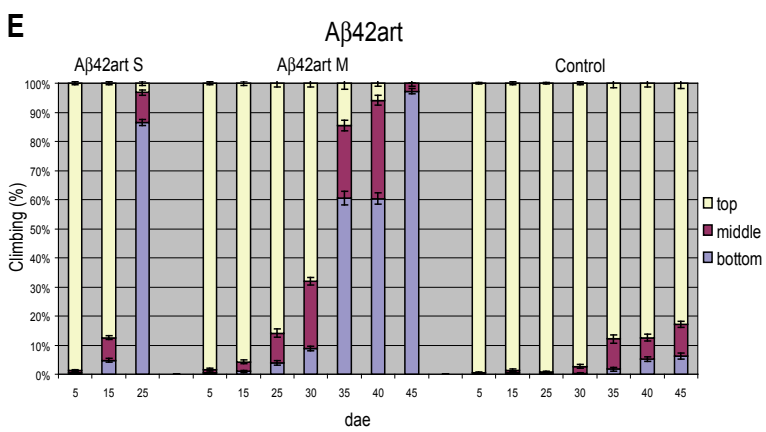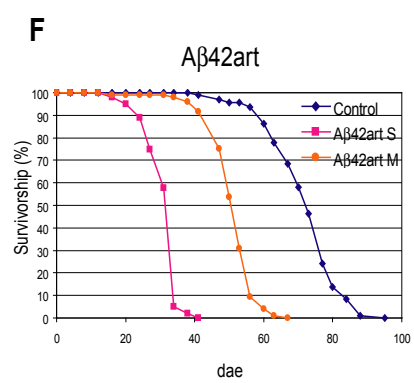

Supplement: Figure S4 — Behavioral defects induced by the expression of Aβ42, Aβ42Arc, and Aβ42art peptides were dose dependent. A, C and E, Locomotor dysfunction in independent transgenic lines (W; weak, M; moderate, S; strong expression) of Aβ42 (A), Aβ42Arc (C), and Aβ42art (E). The percent of flies at the top (yellow), middle (magenta), or bottom (blue) of the vial at 10 seconds after knocking flies to the bottom are shown (average±SEM (n = 10)). B, D and F, The percent survivorship of independent transgenic lines (W, M, and S) of Aβ42 (B), Aβ42Arc (D), and Aβ42art (F) was plotted against the age (dae). The expression levels of Aβ peptides in all transgenic lines are shown in Figure 1B, and indicated as S (strong), M (moderate) or W (weak). The results are summarized in table S1. In the main text, the data from Aβ42 M, Aβ42Arc M and Aβ42art M (asterisks in Figure 1B) are presented. (0.40 MB PDF) [file pone.0001703.s004.pdf]

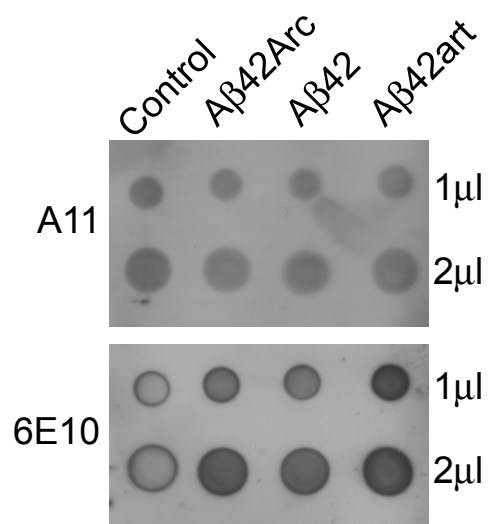

Supplement: Figure S5 — No A11-positive Aβ oligomers in Aβ42, Aβ42Arc, and Aβ42art fly brains. Fly brain lysates (1 or 2 µl) were applied on membrane and probed with 6E10 (top) or oligomer-specific antibody, A11 (bottom). No specific signal was observed with A11 antibody (top, compare Control and Aβ42Arc, Aβ42 or Aβ42art), while 6E10 detected Aβ in fly brains (bottom, compare Control and Aβ42Arc, Aβ42 or Aβ42art). elav-Gal4c155 flies were used as control. (1.61 MB PDF) [file pone.0001703.s005.pdf]

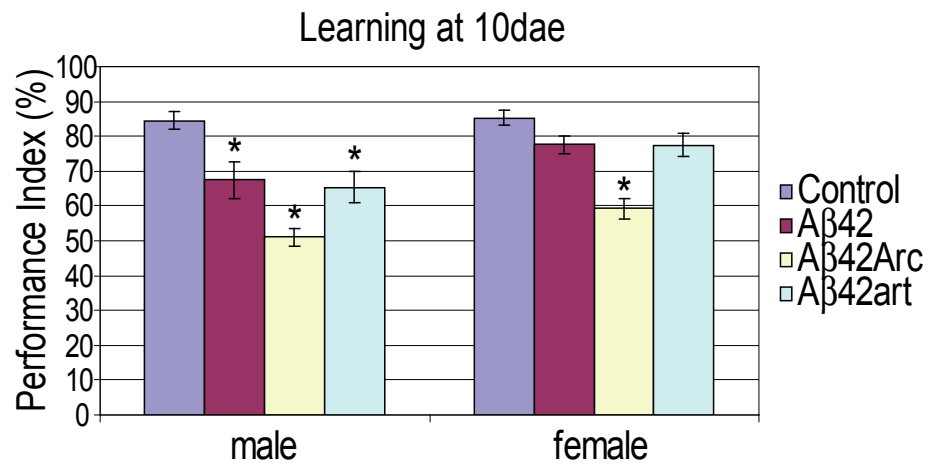

Supplement: Figure S6 — Learning defects in Aβ42, Aβ42Arc, and Aβ42art flies. Learning ability was assessed by Pavlovian olfactory conditioning at 10 dae. Asterisks indicate a significant difference from control (n = 6–8, α<0.05, Tukey-Kramer significant difference). Average learning scores±SEM are shown. (0.04 MB PDF) [file pone.0001703.s006.pdf]

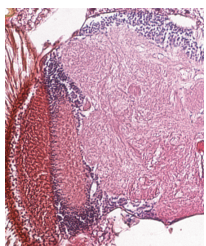

Brain thickness

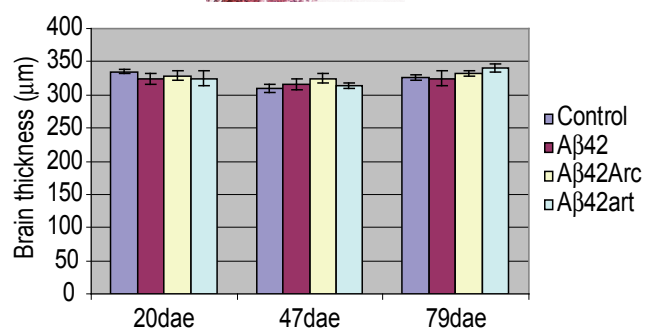

Supplement: Figure S7 — Brain sizes of control, Aβ42, Aβ42Arc, and Aβ42art flies were not significantly different. The thickness of fly brains was measured as indicated, and presented as average±SEM (n = 5 individual flies). The age of the flies is indicated at the bottom. (0.62 MB PDF) [file pone.0001703.s007.pdf]

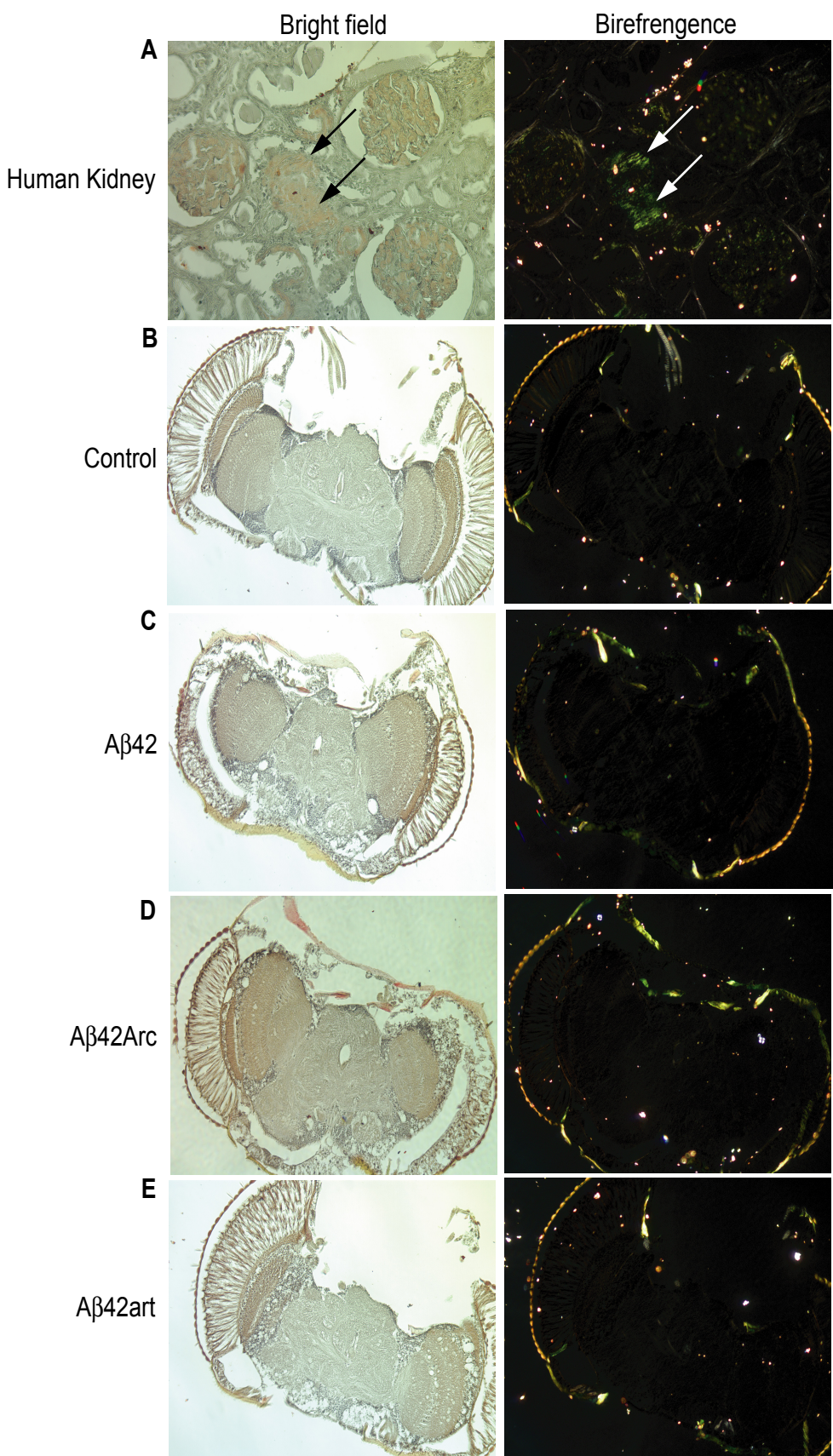

Supplement: Figure S8 — The majority of Aβ aggregates in fly brains are not stained by Congo-Red. Paraffin sections of brains of 25 dae control (B), Aβ42 (C), Aβ42Arc (D) and Aβ42art (E) flies were stained with Congo-Red. Sections from human kidney tissue containing intracellular amyloid were used as a positive control (A). Amyloid was observed as pink signals under bright field, and β-pleated structures were detected as birefringent apple-green signals using a polarizing filter (arrows in (A)). None of Aβ fly brains were stained with Congo-Red (C–E). The numerous vacuoles in (C–E) indicate neurodegeneration. For this analysis, transgenic lines with the highest expression of each Aβ peptide (Aβ42 S, Aβ42Arc M and Aβ42art S) were used. (7.67 MB PDF) [file pone.0001703.s008.pdf]
