# Supplementary material for: Aβ42 Mutants with Different Aggregation Profiles Induce Distinct Pathologies in Drosophila
Source: PLoS One. 2008 Feb 27;3(2):e1703. doi: 10.1371/journal.pone.0001703 (PMC2250771; doi:10.1371/journal.pone.0001703)
Supplement: Table S1 — Summary of the characterization of Aβ transgenic lines. Asterisks (*) indicate transgenic lines primarily used in this study. (0.08 MB PDF) [file pone.0001703.s009.pdf]

Transgenic line	Aβ expression[1)]	Climbing defects (dae)[2)]	Average lifespan (dae)
Aβ42 S	1.89 ± 0.17	25	35.6 (n=111 flies)
**Aβ42 M***	**1.00**	**35**	**46.2 (n=89 flies)**
Aβ42 W	0.44 ± 0.10	40	53.3 (n=93 flies)
Aβ42Arc S(M+W)	1.68	15[†]	21.2 (n=116 flies)
**Aβ42Arc M***	**1.01 ± 0.06**	**25**	**32.9 (n=98 flies)**
Aβ42Arc W	0.67 ± 0.04	35	44.4 (n=99 flies)
Aβ42art S	2.40 ± 0.23	25	31.4 (n=99 flies)
**Aβ42art M***	**1.02 ± 0.05**	**45**	**51.4 (n=97 flies)**
Control	-	-	72.4 (n=95 flies)

[1)] Relative levels of Aβ to Aβ42 M are shown (n=3, average ± SD). [2)] Days-after-eclosion (dae) when climbing disability in 80% of the flies occurred except for[†]. [†] Days-after-eclosion (dae) when climbing disability in 70% of the flies occurred.
