# Supplementary material for: Aβ42 Mutants with Different Aggregation Profiles Induce Distinct Pathologies in Drosophila
Source: PLoS One. 2008 Feb 27;3(2):e1703. doi: 10.1371/journal.pone.0001703 (PMC2250771; doi:10.1371/journal.pone.0001703)
Supplement: Table S2 — Shock reactivity and olfactory acuity of transgenic flies at 10 dae. Aβ42, Aβ42Arc, and Aβ42art flies did not show any significant differences from control flies (n = 6, α<0.05, Tukey-Kramer significant difference). Average scores±SEM are shown. In MCH olfactory acuity of males, there were no differences relative to controls, but Aβ42Arc and Aβ42art flies were significantly different from each other (*). (0.18 MB PDF) [file pone.0001703.s010.pdf]

**Genotype (females)**	**Shock reactivity (60 V)**	**Olfactory acuity (MCH, 10^{-3})**	**Olfactory acuity (OCT, 10^{-3})**
Control	72 ± 4	45 ± 7	31 ± 7
Aβ42	80 ± 4	39 ± 7	24 ± 3
Aβ42Arc	77 ± 6	28 ± 6	29 ± 6
Aβ42art	73 ± 2	49 ± 5	27 ± 5

**Genotype (males)**	**Shock reactivity (60 V)**	**Olfactory acuity (MCH, 10^{-3})**	**Olfactory acuity (OCT, 10^{-3})**
Control	67 ± 5	46 ± 9	23 ± 5
Aβ42	69 ± 5	40 ± 3	21 ± 6
Aβ42Arc	67 ± 3	24 ± 4*	12 ± 4
Aβ42art	68 ± 5	57 ± 10*	17 ± 6
